# Workplace access, burnout, and prescription drug misuse among Korean hospital nurses: a cross-sectional study

**DOI:** 10.1186/s12912-024-02042-4

**Published:** 2024-06-01

**Authors:** Chaehee Kim, Kihye Han, Alison M. Trinkoff, Hyang Baek

**Affiliations:** 1 Graduate School Department of Nursing, Chung-Ang University, Seoul, South Korea; 2 College of Nursing, Chung-Ang University, Seoul, South Korea; 3 University of Maryland School of Nursing, Baltimore, MD USA

**Keywords:** Availability, Burnout, Nurse, Prescription drug misuse, South Korea, Workplace availability

## Abstract

**Background:**

Prescription drug misuse has been identified as a global issue of concern. Nurses’ prescription drug misuse is linked to personal health problems and impaired nursing care. This study explored the level of South Korean hospital nurses’ prescription drug misuse and examined associations with workplace access and burnout.

**Methods:**

This cross-sectional study used data from 1142 nurses working in South Korean hospital settings. Nurses completed the online survey anonymously. Descriptive analysis, logistic regression, and Shapley value assessment were conducted.

**Results:**

Pain relievers (44.2%), IV drips (26.8%), and antibiotics (13.5%) were the most commonly misused drugs among hospital nurses. Accessibility in the workplace was high, with nurses reporting frequent administration of IV drips, pain relievers, and antibiotics and perceiving these drugs as easily accessible. Logistic regression demonstrated that perceived availability was significantly related to misuse across all drug types. Burnout was associated with IV drips, sleeping pills, and steroids, increasing the likelihood of misuse. Shapley feature importance analysis highlighted perceived availability as the most influential factor for IV drips, pain relievers, and steroids, while burnout emerged as crucial for antibiotics and sleeping pills. Notably, age played a significant role in appetite suppressant misuse, distinguishing it from other drugs.

**Conclusion:**

Our results revealed that workplace access and burnout are associated with nurses’ prescription drug misuse. Effective educational strategies are essential for enhancing nurses’ willingness to seek help for personal health issues. Hospital organizations play a crucial role in facilitating access to healthcare and fostering a supportive environment for nurses to seek treatment when necessary. Additionally, governmental policies should prioritize the implementation of active surveillance systems to monitor medication usage in healthcare settings, thereby mitigating drug misuse among healthcare professionals. By addressing these issues, we can ensure the well-being of nurses and promote a safer healthcare environment.

## Background

Prescription drug misuse (defined as the independent use of prescription drugs without or beyond a doctor’s prescription) has been identified as a global issue of concern [1]. Of the estimated 59 million-plus Americans who use illicit drugs, 16 million are estimated to misuse prescription drugs [2]. Prescription drug misuse has increased over the past 15 years in the United States, contributing to a 500% increase in drug overdose-related deaths [3, 4]. Prescription pain medications are the second most misused group of drugs after cannabis [5]. Prescription drug misuse has been linked to increased emergency room visits, overdose deaths, and addiction treatment admissions [6–8]. Prescription drug misuse places an additional burden on individuals and society due to its association with illicit drug use and alcohol use disorders [9]; it has been shown to lead to the use of heroin and other injected drugs [10].

Healthcare professionals face potential exposure to the risk of prescription drug misuse [11–14]. Easy access to medications due to the nature of their job can increase healthcare workers’ vulnerability to prescription drug abuse or misuse [11]. Among healthcare professionals, anesthesia care providers are more likely to develop a substance use disorder than others due to the relatively easy drug access and independent nature of their tasks [12]. In a Dutch study, physicians tended to misuse prescription drugs more than non-physicians [13]. A recent study reported that more nurses misused prescription-type drugs compared with the general population (9.9% vs. 5.9%) [14]. The prevalence of prescription drug misuse among healthcare professionals, especially nurses who frequently interact with patients, is a significant cause for concern, highlighting the need to thoroughly investigate the multiple factors contributing to prescription drug misuse by nurses.

At the environmental level, work-related factors that might be associated with prescription drug misuse among nurses include high accessibility in the workplace, specifically perceived ease of availability, frequent administration, and poor workplace control [15]. A U.S. study reported that nurses were prone to misuse prescription medications such as amphetamines, opioids, sedatives, and tranquilizers, which are easily accessible in their workplaces [15]. Lack of proper monitoring of controlled medications in hospitals and other care settings poses an occupational hazard for nurses [16]. Nurses’ prescription drug misuse has been linked to personal health issues and impaired nursing care [15, 17]. The deliberate illegal distribution of regulated drugs (i.e., drug diversion) has also been noted as an issue among nurses in the United States [11].

At the individual level, burnout should be considered as a potentially influential factor affecting nurses’ prescription drug misuse. Burnout, defined as prolonged emotional stress leading to physical and mental fatigue, has been shown to have a significant impact on nurses’ well-being and coping mechanisms [18, 19]. The demanding nature of their work, irregular shifts, and exposure to traumatic events create an environment ripe for burnout. The chronic stress experienced by nurses may increase the likelihood of their using substances, including prescription drugs, as a way of coping with emotional and mental distress [17].

In Western countries, research efforts have studied the prevalence, etiological factors, and estimated outcomes of nurses’ prescription drug misuse [15]. However, in South Korea, although prescription drug misuse has been investigated in limited studies (e.g., in older persons [20]), there is a lack of research on nurses’ prescription drug misuse. Overseas research may not be generalizable to the Korean context because drug use in South Korea and North America, in general, are quite different; drug use in Asian countries is regarded as a crime rather than a public health problem [21]. According to the latest cross-national report [22], the most recently available data on the prevalence of drug use in Korea, which was collected almost 20 years ago (2004), showed relatively low levels of misuse when compared with 2020 U.S. data: cannabis (0.29% of the population), amphetamines (0.12%), and opioids (0.08%) in Korea vs. cannabis (21.9%), amphetamines (5.65%), and opioids (2.9%) in the United States.

In relation to the high turnover rate among nurses in South Korea, which is about threefold that of other professions (14.5% in 2021), extreme workload and stress levels have been reported as important contributing factors [23–25]. In such circumstances, Korean nurses may be more prone to ignoring their personal health issues or resorting to prescription drug misuse. Therefore, there is an alarming need for research on prescription drug misuse to explore avenues for enhancing the well-being and safety of nurses. As there are no available data or research about prescription drug misuse among Korean nurses, our study was conducted to address this research gap. This study explored the level of nurses’ prescription drug misuse and examined the effects of workplace access and burnout on prescription drug misuse.

## Methods

### Study design

This study employed a quantitative cross-sectional descriptive design. By employing quantitative methods, this study aims to investigate the level of nurses’ prescription drug misuse and examine the effects of workplace access and burnout on prescription drug misuse. We hypothesized that high workplace access and high burnout would be associated with prescription drug misuse among hospital nurses.

### Study setting

This research was part of the Nursing Teamwork Study, designed to assess the work-related and personal characteristics of nurses and their association with nurses’ health outcomes. Anonymous survey data were collected online through a Korean mobile application.

### Population

In line with our research objectives, the target population consisted of experienced nurses working in hospital settings. We included registered nurses [1] who were currently working in hospital settings (e.g., tertiary, long-term care (LTC)/specialty, hospital clinics) with at least 6 months of work experience, and [2] who provided consent to participate in the study.

### Sampling and sample size

Our study recruited Korean hospital nurses through a widely used mobile app via convenience sampling. When conducting a power analysis using G*Power 3.1.9.7 (Heinrich Heine University Düsseldorf, Düsseldorf, Germany), our regression analysis was expected to have data from at least 399 subjects [15]. Our sample size exceeded the minimum requirement, potentially enhancing the accuracy and reliability of the study findings.

### Data collection

Data collection took place in May 2021. Study participants were enlisted through the utilization of ‘My Duty,’ a mobile application used by roughly 85% of Korean nurses. Those currently using the application were extended an invitation to participate in the survey directly through the mobile platform and were able to proceed upon granting consent.

### Measures

#### Prescription drug misuse

Prescription drug misuse in the past year was asked using this single item: “How often have you used the following prescription drugs without a doctor’s prescription in the past year?”, which was adapted from the U.S. national survey on drug use and health [26]. The item has demonstrated satisfactory psychometric properties, e.g., good reliability (kappa 0.65–0.85) and content validity among nurses [15, 27].

For the specific types of prescription drugs, we referred to the substance use items from the Nurses’ Worklife and Wellness Study (NWWS) [14]. Considering the cultural differences between the two countries, we substituted the NWWS misuse items (benzodiazepines, opiates, non-narcotic pain relievers, and stimulants) with frequently misused prescription medications in the general Korean population [28–31]. A list of six types of prescription drugs was provided as follows: IV drips (glucose, normal saline, Hartmann’s solution), pain relievers, antibiotics, sleeping pills, steroids, and appetite suppressants. Participants were informed that the drugs listed in the survey questionnaire were prescription-type drugs, not over-the-counter drugs that can be purchased at supermarkets or pharmacies. The response options were 1 = never, 2 = rarely, 3 = once every 6 months, 4 = once a month, 5 = once a week, and 6 = daily. Based on prior drug-related research, we aggregated the responses into two groups to prioritize the presence or absence of the behavior over its frequency: yes (2 = rarely to 6 = daily), which indicated any use reported last year, or no (1 = never) [15, 32, 33].

#### Accessibility of drugs in the workplace

For our investigation of the accessibility of drugs in the workplace, we used the NWWS items, which cover two dimensions: frequency of administration and perceived availability. These showed good construct and content validity in a nurse population (e.g., factor loadings 0.86 to 0.89) [15]. To assess the frequency of administration of the six medication types, nurses were asked, “How often do you administer the following drugs to patients in your work?” with a 4-point response option of 1 = never, 2 = rarely, 3 = sometimes, and 4 = often. We then aggregated the responses into three groups: never, rarely/sometimes, or often [15]. To assess the perceived availability of the six medication types, nurses were asked, “How easy is it to get the following drugs from your workplace for your own purposes?” with response options ranging from 1 = very difficult, 2 = difficult, 3 = easy, and 4 = very easy.

#### Burnout

Burnout was assessed using the Korean version of the Copenhagen Burnout Inventory (K-CBI) [34, 35]. The K-CBI comprises 19 items assessed on a 5-point Likert scale (1 = never/almost never, 2 = rarely, 3 = occasionally, 4 = frequently, 5 = always). Scale scores were computed as the average value of the items, with higher scores indicating increased burnout levels. The K-CBI has demonstrated acceptable content and construct validity along with good internal consistency reliability among Korean nurses [36]. In our sample, the K-CBI was shown to be reliable (Cronbach’s alpha = 0.94).

### Data analysis

SPSS Statistics for Windows, version 26.0 (IBM Corp., Armonk, NY, USA) and R, version 4.3.1 (R Foundation for Statistical Computing, Vienna, Austria), were used for statistical analyses. To explore the level of nurses’ prescription drug misuse and workplace access, frequencies and percentages were calculated. The level of burnout was evaluated using its mean and standard deviation. To examine the effects of workplace access and burnout on prescription drug misuse, logistic regression models were created. For the statistical analysis, we inspected the assumptions of the regression analysis. Firstly, the observations were independent, as the data were collected online from non-overlapping individuals without nested features. Secondly, no significant collinearity among the variables was found (all VIF values < 1.30). Lastly, visual inspection of the data revealed no outliers. Logistic regression models were adjusted for potential confounders of personal characteristics such as age and gender. Variables with an odds ratio (OR) exceeding 1 signified an increased likelihood of misusing prescription drugs. We hypothesized that workplace access and burnout would have odds ratios higher than 1 for prescription drug misuse. The significance level was set at 95%.

To quantitatively evaluate the relative importance of explanatory variables for nurses’ prescription drug misuse, Shapley values were computed [37]. By measuring each variable’s contribution to the model’s prediction, Shapley values offer insights into how individual variables influence the overall prediction. Global Shapley plots visually summarize variable importance in a predictive model, with longer bars indicating higher average Shapley values and showcasing the features’ impact on predictions. Positive or negative extensions reveal the direction of influence. Comparing bar lengths helps rank features, offering insights into their relative importance and interaction effects.

### Ethical considerations

This research was approved by the Institutional Review Board of Chung-Ang University (IRB No. 1041078-202203-HR-114). This research adhered to guidelines and standards. Participation in this study was voluntary, and informed consent to participate was obtained from all participants. After providing an introduction and explanation about the study aims and procedures, only nurses who confirmed their eligibility and agreement to participate in the study voluntarily accessed the online survey.

## Results

### Sample characteristics

Out of 1901 individuals who accessed the study link, 1759 consented to participate in the study, and among them, 1160 completed the survey (response rate = 61%). Subsequently, 18 respondents were further excluded for the following reasons: not a registered nurse (*n* = 1), less than 6 months of experience (*n* = 3), and not working in hospital settings (*n* = 14), resulting in a final sample of 1142 nurses. Sociodemographic and work characteristics of the sample are presented elsewhere [38]. Briefly, the nurse participants were predominantly female (93.5%) with a mean age of 29 years, and the majority of them were not married (82.0%). The most common specialty was general wards (e.g., adult care such as medical-surgical) (60.5%), followed by intensive care unit (ICU)/neonatal intensive care unit (NICU) (18.8%) and emergency room (ER) (7.4%). Most nurses worked on a rotating schedule, including night shifts (87.7%); 2% of them worked only night shifts. The average level of burnout among the nurses was 3.26 (SD = 0.77, range 1–5).

### Prescription drug misuse among nurses

Pain relievers (44.2%), IV drips (glucose, normal saline, Hartmann’s solution) (26.8%), and antibiotics (13.5%) were the most misused types of drugs (Table 1), with sleeping pills (8.1%), steroids (8.1%), and appetite suppressants (5.4%) less commonly misused.

**Table 1:** Frequency of prescription drug misuse^a^ among nurses in the past year

Type of Medication		*n*	%
IV drips	yes	306	26.8
	no	836	73.2
Pain relievers	yes	505	44.2
	no	637	55.8
Antibiotics	yes	154	13.5
	no	988	86.5
Sleeping pills	yes	93	8.1
	no	1049	91.9
Steroids	yes	93	8.1
	no	1049	91.9
Appetite suppressants	yes	62	5.4
	no	1080	94.6

Note. ^a^Prescription drug misuse refers to independent prescription drug use without or beyond a doctor’s prescription in the past year

### Accessibility to drugs at the workplace

Most nurses reported frequently administering IV drips (84.8% reported often), pain relievers (80.1%), and antibiotics (80.0%) in their workplace (Table 2). Nurses perceived that IV drips (68.3%), pain relievers (66.5%), and antibiotics (40.4%) were easily accessible at the workplace if they sought them for their own use.

**Table 2:** Accessibility to prescription drugs at the workplace

Frequency of administration^a^		*n*	%	Perceived availability^b^		*n*	%
IV drips	Often	968	84.8	IV drips	Very easy	365	32.0
	Rarely/Sometimes	110	9.6		Easy	415	36.3
	Never	64	5.6		Difficult	178	15.6
					Very difficult	184	16.1
Pain relievers	Often	915	80.1	Pain relievers	Very easy	265	23.2
	Rarely/Sometimes	180	15.8		Easy	495	43.3
	Never	47	4.1		Difficult	196	17.2
					Very difficult	186	16.3
Antibiotics	Often	914	80.0	Antibiotics	Very easy	166	14.5
	Rarely/Sometimes	147	12.9		Easy	296	25.9
	Never	81	7.1		Difficult	278	24.3
					Very difficult	402	35.2
Sleeping pills	Often	430	33.7	Sleeping pills	Very easy	44	3.9
	Rarely/Sometimes	556	48.7		Easy	186	16.3
	Never	156	13.7		Difficult	326	28.5
					Very difficult	586	51.3
Steroids	Often	420	36.8	Steroids	Very easy	93	8.1
	Rarely/Sometimes	578	50.6		Easy	230	20.1
	Never	144	12.6		Difficult	345	30.2
					Very difficult	474	41.5
Appetite suppressants	Often	23	2.0	Appetite suppressants	Very easy	18	1.6
	Rarely/Sometimes	174	15.2		Easy	80	7.0
	Never	945	82.7		Difficult	278	24.3
					Very difficult	766	67.1

Note. ^a^Frequency of administration: “How often do you administer the following drugs to patients at your work?”

^b^Perceived availability: “How easy is it to get the following drugs from your workplace for your own purposes?”

### Effects of workplace accessibility and burnout with nurses’ prescription drug misuse

Logistic regression analysis revealed that perceived availability was significantly related to all types of prescription drug misuse (Table 3). For example, nurses who reported that it was very easy to obtain IV drips for personal use in the workplace had 9.91 times higher odds of misusing IV drips compared to those who found it very difficult (OR = 9.91, CI = 5.16–19.06). Frequency of administration was significantly associated with IV drips, pain relievers, and appetite suppressants: nurses who often administer IV drips were three times more likely to misuse IV drips than nurses who do not administer IV drips (OR = 3.00, CI = 1.16–7.80). The odds of misuse among nurses who often administered appetite suppressants were 3.79 times higher than among those who never administered appetite suppressants (OR = 3.79, CI = 1.18–12.13).

Burnout was significantly associated with IV drips, sleeping pills, and steroids. Nurses were 38%, 62%, and 53% more likely to misuse IV drips, sleeping pills, and steroids, respectively, for each unit increase in burnout (OR = 1.38, CI = 1.15–1.66 for IV drips; OR = 1.62, CI = 1.20–2.19 for sleeping pills; OR = 1.53, CI = 1.14–2.07 for steroids).

For antibiotics, sleeping pills, steroids, and appetite suppressants, nurses were 7–8% more likely to misuse these drugs for every 1-year increase in age (OR = 1.07, CI = 1.03–1.10 for antibiotics; OR = 1.07, CI = 1.03–1.11 for sleeping pills; OR = 1.08, CI = 1.04–1.12 for steroids; OR = 1.08, CI = 1.03–1.12 for appetite suppressants). In contrast, nurses had 3% lower odds of misusing IV drips with each year of age increase (OR = 0.97, CI = 0.93-1.00).

**Table 3:** Associations of workplace accessibility, burnout, and individual factors with nurses’ prescription drug misuse^a^

		IV drips		Pain relievers		Antibiotics		Sleeping pills		Steroids		Appetite suppressants	
		OR	95% CI	OR	95% CI	OR	95% CI	OR	95% CI	OR	95% CI	OR	95% CI
Frequency of administration^b^	Often	3.00^*^	1.16, 7.80	1.88	0.92, 3.85	0.95	0.45, 1.99	1.66	0.74, 3.73	1.00	0.44, 2.28	3.79^*^	1.18, 12.13
	Rarely/Sometimes	4.39^**^	1.54, 12.52	2.31^*^	1.08, 4.95	1.80	0.79, 4.09	1.23	0.55, 2.75	1.40	0.63, 3.08	1.85	0.99, 3.47
	Never (ref)	1.00		1.00		1.00		1.00		1.00		1.00	
Perceived availability^c^	Very easy	9.91^***^	5.16, 19.06	4.74^***^	3.08, 7.32	3.17^***^	1.82, 5.51	1.99	0.71, 5.56	4.32^***^	1.94, 9.64	6.01^**^	1.79, 20.22
	Easy	6.46^***^	3.37, 12.39	3.05^***^	2.06, 4.51	2.92^***^	1.80, 4.72	2.81^***^	1.59, 4.98	4.57^***^	2.51, 8.32	2.94^*^	1.29, 6.68
	Difficult	2.66^**^	1.27, 5.58	1.57	0.99, 2.48	1.88^*^	1.12, 3.14	1.92^*^	1.12, 3.28	1.90^*^	1.02, 3.53	1.59	0.86, 2.95
	Very difficult (ref)	1.00		1.00		1.00		1.00		1.00		1.00	
Age		0.97^*^	0.93, 1.00	1.02	0.99, 1.04	1.07^***^	1.03, 1.10	1.07^***^	1.03, 1.11	1.08^***^	1.04, 1.12	1.08^***^	1.03, 1.12
Gender	Female	0.8	0.44, 1.37	1.10	0.67, 1.83	1.06	0.51, 2.20	0.80	0.35, 1.82	1.87	0.56, 6.18	1.31	0.39, 4.39
	Male (ref)	1.00		1.00		1.00		1.00		1.00		1.00	
Burnout		1.38^***^	1.15, 1.66	1.16	0.99, 1.36	1.21	0.96, 1.52	1.62^**^	1.20, 2.19	1.53^**^	1.14, 2.07	1.25	0.89, 1.76

Note. ***p < 0.001, **p < 0.01, *p < 0.05

^a^Prescription drug misuse refers to self-directed prescription drug use without or beyond a doctor’s prescription in the past year

^b^Frequency of administration: “How often do you administer the following drugs to patients at your work?”

^c^Perceived availability: “How easy is it to get the following drugs from your workplace for your own purposes?”

### Feature importance of mean absolute Shapley values

**Fig. 1:**
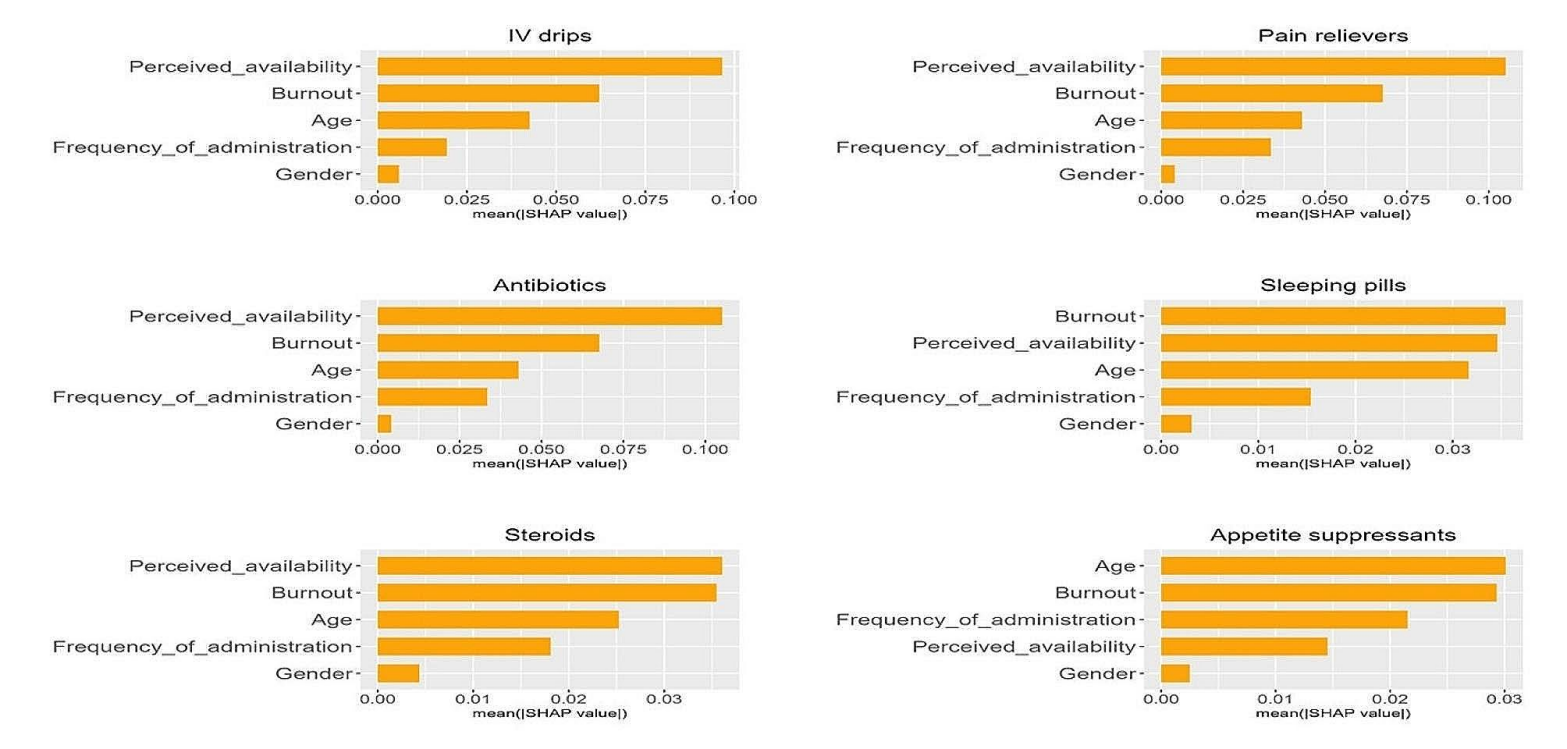
Feature importance of mean absolute Shapley values

The variables were ranked in descending order of their impact, with the most significant influence at the top (Fig. 1). Through the analysis of Shapley feature importance in the predictive model for prescription drug usage, perceived availability emerged as the most influential factor for IV drips, pain relievers, and steroids, and closely followed the top factor for antibiotics and sleeping pills. Burnout surfaced as the most influential feature for antibiotics and sleeping pills, and as the second most influential feature for the other four drugs (IV drips, pain relievers, steroids, and appetite suppressants). For appetite suppressants, distinct from other drugs, age stood out as the most influential feature impacting drug misuse, with younger nurses being more likely to misuse them. Gender exhibited the lowest impact across all medications.

## Discussion

To the best of our knowledge, this is the first study to investigate the level of prescription drug misuse among nurses in South Korea. Considering the strict Korean social norms regarding substance use and to obtain accurate responses, we conducted an anonymous online survey on nurses recruited through a mobile shift-work scheduler application that is frequently used by nurses. Our study identified significant effects of workplace accessibility (at the environmental level) and burnout (at the individual level) on prescription drug misuse among nurses. These findings provide evidence to guide the formulation of targeted educational and policy interventions to address these interconnected factors within the nursing profession.

Although the level of drug misuse by nurses had not been previously assessed in South Korea, U.S. nurses have been reported to misuse prescription drugs at higher rates than the general population. Given nurses’ experience with administering drugs to patients, they may feel capable of controlling and monitoring their drug use for personal needs and eventually decide to use drugs without a doctor’s prescription [39]. Moreover, nurses commonly perceive that health issues can be treated by taking prescription drugs on their own without the need to visit a doctor, thus saving money and time [40]. However, the misuse of prescription drugs can result in drug dependence and adverse health effects when administered without consulting an expert [8]. This behavior not only endangers nurses’ well-being but also undermines patient trust in healthcare providers. This erosion of trust has far-reaching implications, tarnishing the image of the nursing profession as a whole.

The prescription drugs commonly used by nurses (pain relievers, IV drips, and antibiotics) reflect their professional challenges. The physically demanding nature of their tasks, coupled with irregular shift patterns and exposure to high-stress environments, leads nurses to turn to these medications for health challenges. Complaints of persistent back pain and musculoskeletal conditions among nurses underscore the toll of physically strenuous work demands [41]. Moreover, studies reveal a higher prevalence of health issues among those working rotating shifts due to circadian clock changes, which negatively impact various body systems [42, 43]. Lack of timely or convenient healthcare services when needed might lead to drug self-prescription and misuse among healthcare workers [44, 45]. In Korea, only 2% of pharmacies are accessible at night [46], highlighting the need for improved healthcare services for shift workers. Hospitals should establish targeted occupational health programs for nurses. Nurse managers should closely monitor their staff for any health problems, deteriorating work performance (e.g., absenteeism and burnout), or inappropriate access to prescription-type drugs [42, 47].

In our study, the relatively high level of misuse of IV drips was of concern. As little is known about the misuse of IV drips, it is difficult to fully explain its scope or contributing factors. Considering the circumstances, IV drip administration is an invasive procedure that requires needles and an aseptic technique. In a non-therapeutic, non-sterile environment for self-medication, undesirable complications (e.g., infections and skin damage) could occur; nurses may feel that they have the knowledge to prevent these adverse outcomes [48]. The phenomenon of IV drip use is not fully understood, and its contributing factors should be investigated in future studies.

At the environmental level, our study revealed that increased workplace accessibility (i.e., frequency of administration and perceived availability) of prescription drugs at the workplace is linked to a higher likelihood of misuse, consistent with existing research [15]. Easy access to drugs, including drug administration and access to controlled medication cabinets, as well as drug wastage within healthcare settings, increases the risk of healthcare professionals misusing or diverting medications for personal use [40, 42, 45]. Drug diversion, particularly the unauthorized use of prescription medications for self-administration, has emerged as a great concern in the healthcare industry [11]. Drug diversion is not only a criminal offense but also profoundly detrimental to both patients and healthcare providers [11]. Patients might be deprived of essential treatments, hindering their recovery and overall well-being [49]. Financially, the cost would incur for diverted medication not used for intended patient care leads to unwarranted expenses. Monitoring systems for drug counts should be implemented to reduce the perceived availability of drugs among nurses. For example, an electronic controlled substance ordering system (CSOS) could be used instead of a traditional paper form [50].

At the individual level, burnout also had a significant impact on prescription drug misuse, with higher levels of burnout associated with higher levels of misuse. This is consistent with previous research that found higher levels of burnout were associated with unprofessional behaviors such as substance use and malpractice [51]. Considering the inherently stressful nature of the nursing profession, particularly during the COVID-19 pandemic in 2020 when our study was conducted, nurses experienced significant exhaustion, leading to burnout [52]. The link between burnout and prescription drug misuse among nurses highlights the imperative for healthcare institutions to proactively address the mental health challenges faced by their staff. Our findings underscore the critical need for institutional efforts emphasizing psychological education and providing robust support systems to mitigate burnout and reduce the likelihood of prescription drug misuse [53].

Our study has strengths as one of the few investigations on prescription drug misuse among nurses in South Korea, and it holds the advantage of examining the relationship between nurses’ burnout, workplace access, and prescription drug misuse, which can contribute to their well-being. However, these findings should be interpreted in consideration of the study’s limitations. First, as we recruited a convenience sample of survey volunteers online, our participants may not be representative of the overall Korean nurse population. It is likely that nurses who were more comfortable with online environments responded to the survey, which could explain the relatively young age of the sample. Second, as the data were based on the participants’ self-reports, there might be social-desirability, recall, denial, or deception effects [54]. Nevertheless, considering that drug-related behaviors are sensitive issues, self-report questionnaires are the most convenient and widely used method for drug use assessments [55], and the online survey allowed us to obtain more accurate and honest information by ensuring anonymity [56]. Since prescription drug misuse and workplace accessibility have not been explored in the Korean nursing population, there may be insufficient evidence for the validity and reliability of the study instruments among Korean hospital nurses. Thorough psychometric testing of the items is warranted in the future. Additionally, data collection occurred during the height of the COVID-19 pandemic, which may have influenced responses such as burnout. Therefore, continuous research efforts are warranted.

## Conclusion

As this is the first study of prescription drug misuse among Korean nurses, it offers baseline information for further research, as the willingness of nurses to report such information was not previously known. Our findings suggest that educational approaches to support the nurses’ help-seeking behavior towards personal/medical care issues should be provided early. This is especially important in South Korea, where workers with health issues reportedly face fear of stigma or other workplace disadvantages that can impede health-promoting behaviors. To reduce nurses’ perceived availability of medication for misuse in the workplace, government-level policies should be implemented to monitor drug counts in units and hospitals. Evidence-based workplace health promotion programs should be provided to increase workers’ stress management capability and health responsibility. In addition, a supportive climate should be created to help nurses access appropriate treatment with confidentiality. Hospitals ought to create specialized occupational health initiatives tailored specifically for nurses so that they can get proper treatment when needed, such as offering medical services during non-standard work hours for shift nurses.

## Data Availability

Data available on request due to privacy/ethical restrictions.

## References

[1] Schepis TS, Klare DL, Ford JA, McCabe SE (2020). Prescription drug misuse: taking a lifespan perspective. Subst Abuse.

[2] National Center for Drug Abuse Statistics. Prescription drug abuse statistics. 2022. https://drugabusestatistics.org/prescription-drug-abuse-statistics/ (accessed 09 June 2022).

[3] National Center for Drug Abuse Statistics. Drug abuse statistics. 2022. https://drugabusestatistics.org/ (accessed 09 June 2022).

[4] Centers for Disease Control and Prevention. National Center for Health Statistics WONDER online database. Multiple cause of death 1999–2000. 2021. https://wonder.cdc.gov/wonder/help/mcd.html/ (accessed 10 January 2022).

[5] Substance Abuse and Mental Health Services Administration. Key substance use and mental health indicators in the United States: results from the 2021 National Survey on Drug Use and Health. 2022. https://www.samhsa.gov/data/report/2021-nsduh-annual-national-report/ (accessed 23 April 2024).

[6] Ford JA, Schepis TS, McCabe SE (2021). Poly-prescription drug misuse across the life course: prevalence and correlates across different adult age cohorts in the U.S. Int J Drug Policy.

[7] Centers for Disease Control and Prevention. Wide-ranging online data for epidemiologic research (WONDER) online database. Underlying cause of death 1999–2000. 2023. https://wonder.cdc.gov/wonder/help/ucd.html/ (accessed 23 April 2024).

[8] National Institute on Drug Abuse. Misuse of prescription drugs research report. 2022. https://nida.nih.gov/publications/research-reports/misuse-prescription-drugs/overview/ (accessed 10 June 2022).

[9] Papp LM, Barringer A, Blumenstock SM, Gu P, Blaydes M, Lam J (2020). Development and acceptability of a method to investigate prescription drug misuse in daily life: ecological momentary assessment study. JMIR Mhealth Uhealth.

[10] Hawangchu D, Rene Lamy F, Stephan Felix M, Phukao D (2022). Transition from nonmedical prescribed opioids to non-injection heroin use among young integrated Thai male users in Bangkok. J Ethn Subst Abuse.

[11] Perry JC, Vandenhouten CL (2019). Drug diversion detection. Nurs Manage.

[12] Bryson EO (2020). The impact of chemical dependency on health care professionals involved with the delivery of anesthesia. Int Anesthesiol Clin.

[13] Anderson DM, Diris R, Montizaan R, Rees DI. The effect of becoming a physician on prescription drug use. VoxEU. org–CEPR’s policy portal. 2022. https://cepr.org/voxeu/columns/effect-becoming-physician-prescription-drug-use (accessed 2 May 2024).

[14] Trinkoff AM, Selby VL, Han K, Baek H, Steele J, Edwin HS (2022). The prevalence of substance use and substance use problems in registered nurses: estimates from the Nurse Worklife and Wellness Study. J Nurs Regul.

[15] Trinkoff AM, Selby VL, Baek H, Storr CL, Steele J, Han K (2022). Workplace exposures and prescription drug misuse among nurses. J Nurs Adm.

[16] Fauteux N (2022). Are impaired nurses getting the help they need?. Am J Nurs.

[17] Foli KJ, Reddick B, Zhang L, Krcelich K. Substance use in registered nurses: I heard about a nurse who… J Am Psychiatr Nurses Assoc. 2020;26(1):65–76. 10.1177/1078390319886369. PMID:31747853. 10.1177/1078390319886369 31747853

[18] Woo T, Ho R, Tang A, Tam W (2020). Global prevalence of burnout symptoms among nurses: a systematic review and meta-analysis. J Psychiatr Res.

[19] Nápoles J (2022). Burnout: a review of the literature. Update. Update Appl Res Music Educ.

[20] Lee EH, Park JO, Cho JP, Lee CA (2021). Prioritising risk factors for prescription drug overdose among older adults in South Korea: a multi-method study. Int J Environ Res Public Health.

[21] Lataire Q, Peters K, Stoicescu C (2022). Compulsory drug treatment and rehabilitation, health, and human rights in Asia. Health Hum Rights.

[22] United Nations Office on Drugs and Crime. Drug use & treatment. 2022. https://dataunodc.un.org/dp-drug-use-prevalence/ (accessed 20 May 2022).

[23] Hospital Nurses Association. Hospital Nursing Society business report: survey of development status in hospital nurses. 2021. https://khna.or.kr/home/pds/utilities.php?bo_table=board1&wr_id=8072/ (accessed 25 April 2024).

[24] Park JY, Hwang JI (2021). [Relationships among non-nursing tasks, nursing care left undone, nurse outcomes and medical errors in integrated nursing care wards in small and medium-sized general hospitals]. J Korean Acad Nurs.

[25] Lee EK, Kim JS (2020). Nursing stress factors affecting turnover intention among hospital nurses. Int J Nurs Pract.

[26] Substance Abuse and Mental Health Services Administration. Key substance use and mental health indicators in the United States: results from the 2022 National Survey on Drug Use and Health. 2023. https://www.samhsa.gov/data/report/2022-nsduh-annual-national-report/ (accessed 24 April 2024).

[27] Substance Abuse and Mental Health Services Administration. Reliability of key measures in the National Survey on Drug Use and Health. 2010. https://www.samhsa.gov/data/sites/default/files/2k6ReliabilityP/2k6ReliabilityP.pdf/ (accessed 26 April 2024). 30199182

[28] Go SI, Won YW, Kang JH (2022). Safe use of opioids. J Korean Med Assoc.

[29] Jeong HJ, Park JS (2023). Current status and perception of abused drugs by gender among young people. J Ind Convergence.

[30] Shin Y (2015). Fluid therapy as complementary and alternative medicine: an anthropological study about a local medical practice. Inst Cross-Cult Stud.

[31] Heo JY (2023). Antimicrobial stewardship program focused on prolonged carbapenem prescription. Korean J Healthc Assoc Infect Control Prev.

[32] Tam CC, Qiao S, Garrett C, Zhang R, Aghaei A, Aggarwal A (2023). Substance use, psychiatric symptoms, personal mastery, and social support among COVID-19 long haulers: a compensatory model. PLoS ONE.

[33] Kristjansson SD, Agrawal A, Lynskey MT, Chassin LA (2012). Marijuana expectancies and relationships with adolescent and adult marijuana use. Drug Alcohol Depend.

[34] Kristensen TS, Borritz M, Villadsen E, Christensen KB (2005). The Copenhagen Burnout Inventory: a new tool for the assessment of burnout. Work Stress.

[35] Ham MJ. Path analysis of emotional labor and burnout of nurses. [master’s thesis]. Jinju (KR): Gyeongsang University; 2011.

[36] La IS, Yun EK (2019). Effects of trait anger and anger expression on job satisfaction and burnout in preceptor nurses and newly graduated nurses: a dyadic analysis. Asian Nurs Res (Korean Soc Nurs Sci).

[37] Shapley LS, Kuhn H, Tucker A (1953). A value for n-person games. Contributions to the theory of games II.

[38] Ju J, Han K, Ryu J, Cho H (2022). Nurses’ attitudes toward antimicrobial stewardship in South Korea. J Hosp Infect.

[39] Jassim UT, Ebrahim SM (2021). Self-medication among nurses working in Basra teaching hospitals, Iraq. Turk J Physiother Rehabil.

[40] Khatony A, Soroush A, Andayeshgar B, Abdi A (2020). Nursing students’ perceived consequences of self-medication: a qualitative study. BMC Nurs.

[41] Gilchrist A, Pokorná A (2021). Prevalence of musculoskeletal low back pain among registered nurses: results of an online survey. J Clin Nurs.

[42] Cousin L, Roucoux G, Petit AS, Baumann-Coblentz L, Torrente OR, Cannafarina A (2022). Perceived stigma, substance use and self-medication in night-shift healthcare workers: a qualitative study. BMC Health Serv Res.

[43] Voigt RM, Forsyth CB, Keshavarzian A (2019). Circadian rhythms: a regulator of gastrointestinal health and dysfunction. Expert Rev Gastroenterol Hepatol.

[44] Fekadu G, Dugassa D, Negera GZ, Woyessa TB, Turi E, Tolossa T (2020). Self-medication practices and associated factors among health-care professionals in selected hospitals of Western Ethiopia. Patient Prefer Adherence.

[45] Murugan R, Padmavathi T, Hansi BH (2023). Analysis of self-medication practices and patterns among the healthcare professionals in a tertiary care hospital. Panacea J Med Sci.

[46] Health Insurance Review Assessment. Current status of hospitals and pharmacies nationwide. 2021. https://www.hira.or.kr/ (accessed 09 December 2021).

[47] Haile KK, Asnakew S, Waja T, Kerbih HB (2019). Shift work sleep disorders and associated factors among nurses at federal government hospitals in Ethiopia: a cross-sectional study. BMJ Open.

[48] Marks LR, Nolan NS, Liang SY, Durkin MJ, Weimer MB (2022). Infectious complications of injection drug use. Med Clin North Am.

[49] Fitzsimons MG, de Sousa GS, Galstyan A, Quintão VC, Simões CM (2023). Prevention of drug diversion and substance use disorders among anesthesiologists: a narrative review. Braz J Anesthesiol.

[50] Clark J, Fera T, Fortier C, Gullickson K, Hays A, Murdaugh L (2022). ASHP guidelines on preventing diversion of controlled substances. Am J Health Syst Pharm.

[51] Dyrbye LN, West CP, Leep Hunderfund A, Johnson P, Cipriano P, Peterson C (2020). Relationship between burnout and professional behaviors and beliefs among US nurses. J Occup Environ Med.

[52] Galanis P, Vraka I, Fragkou D, Bilali A, Kaitelidou D (2021). Nurses’ burnout and associated risk factors during the COVID-19 pandemic: a systematic review and meta-analysis. J Adv Nurs.

[53] Melnyk BM, Hsieh AP, Davidson J, Carpenter H, Choflet A, Heath J (2021). Promoting nurse mental health. Am Nurse J.

[54] Paulhus DL, Vazire S, Robins RW, Fraley RC, Krueger RF (2007). The self-report method. Handbook of research methods in personality psychology.

[55] Bharat C, Webb P, Wilkinson Z, McKetin R, Grebely J, Farrell M (2023). Agreement between self-reported illicit drug use and biological samples: a systematic review and meta-analysis. Addiction.

[56] Dillman DA (2011). Mail and internet surveys: the tailored design method—2007 update.

